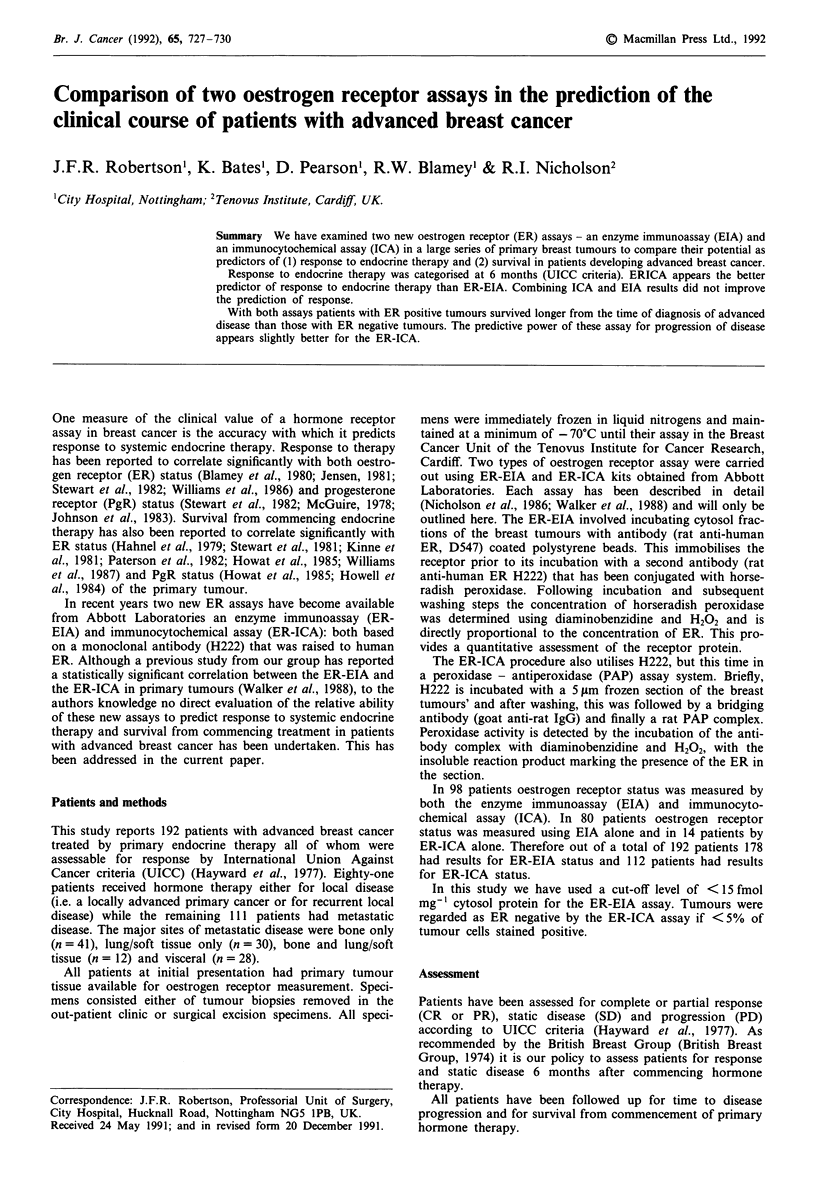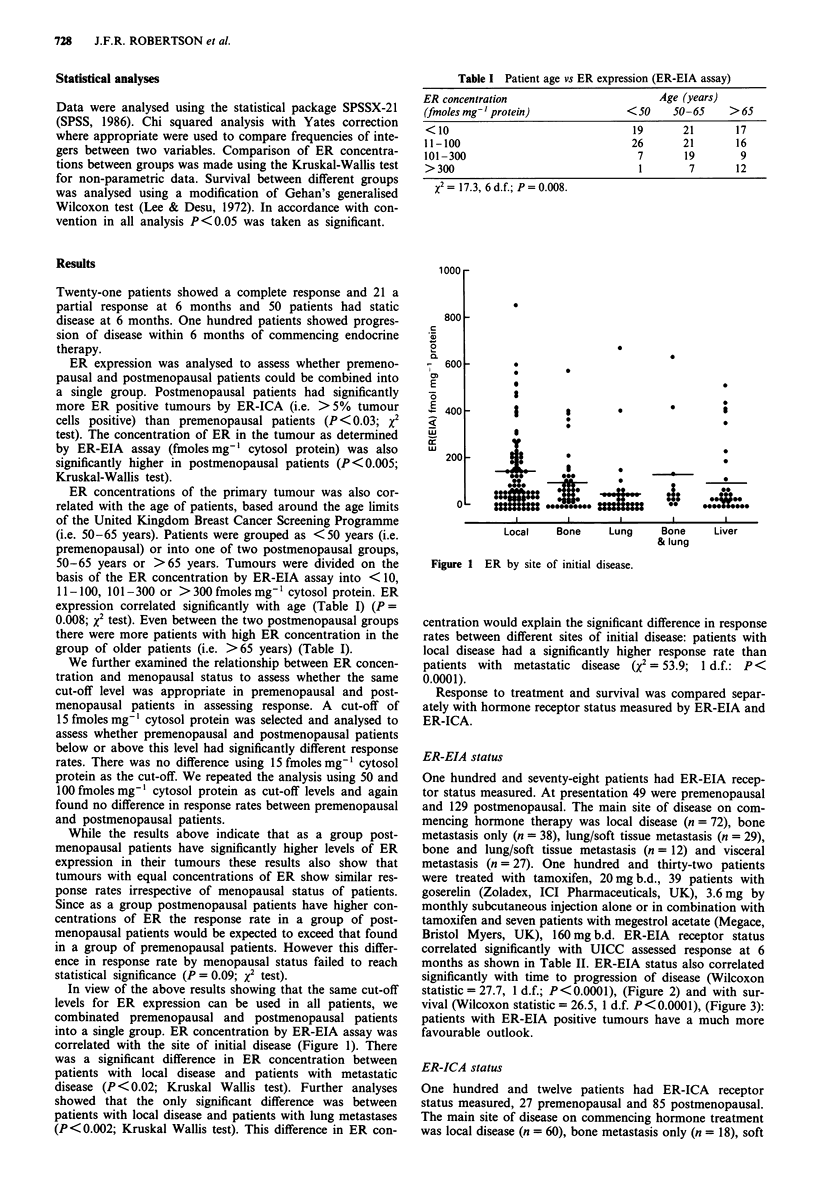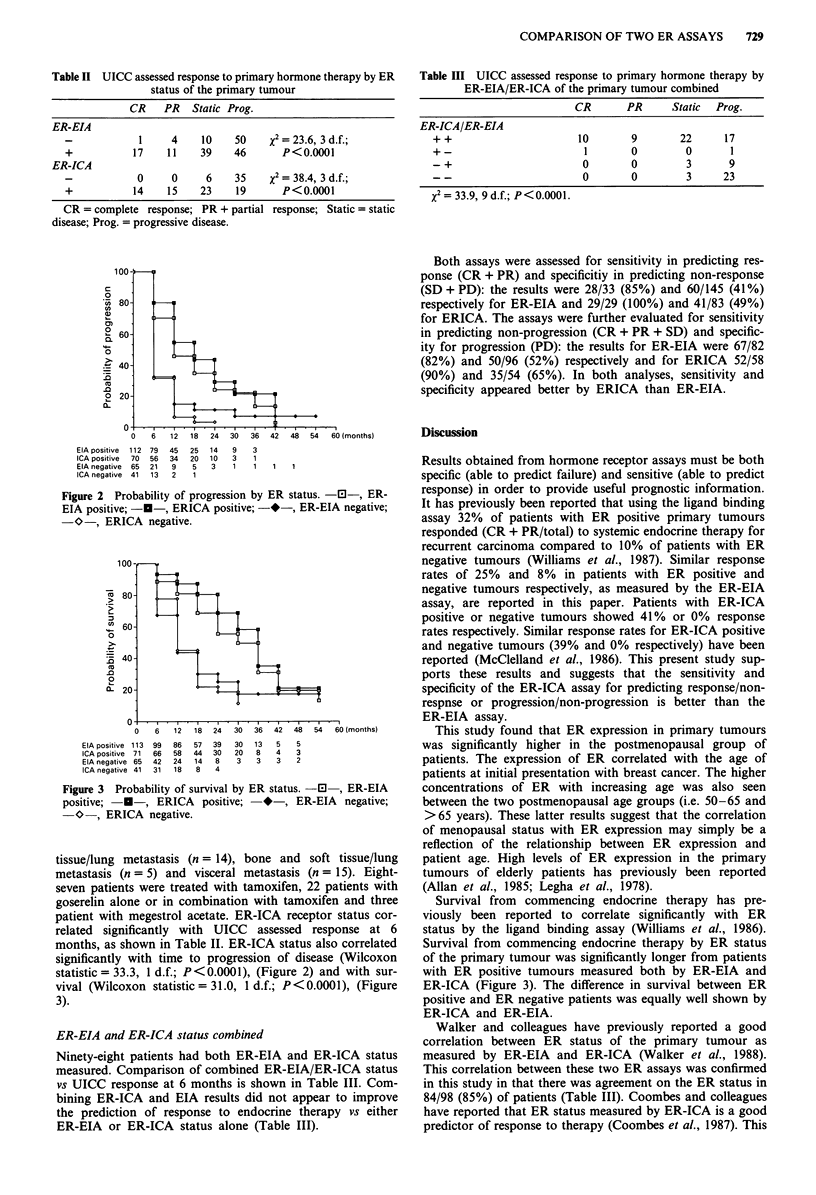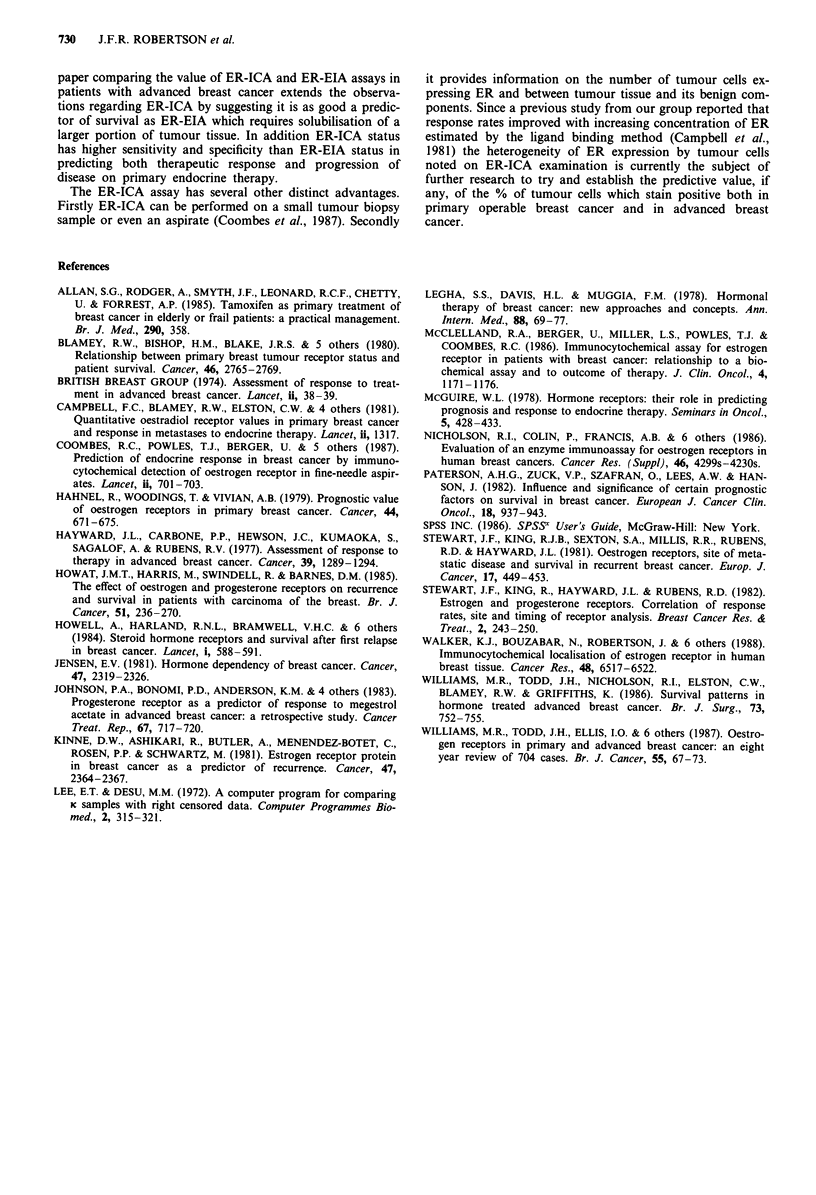# Comparison of two oestrogen receptor assays in the prediction of the clinical course of patients with advanced breast cancer.

**DOI:** 10.1038/bjc.1992.153

**Published:** 1992-05

**Authors:** J. F. Robertson, K. Bates, D. Pearson, R. W. Blamey, R. I. Nicholson

**Affiliations:** City Hospital, Nottingham, UK.

## Abstract

We have examined two new oestrogen receptor (ER) assays--an enzyme immunoassay (EIA) and an immunocytochemical assay (ICA) in a large series of primary breast tumours to compare their potential as predictors of (1) response to endocrine therapy and (2) survival in patients developing advanced breast cancer. Response to endocrine therapy was categorised at 6 months (UICC criteria). ER-ICA appears the better predictor of response to endocrine therapy than ER-EIA. Combining ICA and EIA results did not improve the prediction of response. With both assays patients with ER positive tumours survived longer from the time of diagnosis of advanced disease than those with ER negative tumours. The predictive power of these assay for progression of disease appears slightly better for the ER-ICA.


Br. J. Cancer (1992), 65, 727-730 © Macmillan Press Ltd., 1992

### Comparison of two oestrogen receptor assays in the prediction of the clinical course of patients with advanced breast cancer

J.F.R. Robertson[1], K. Bates[1], D. Pearson[1], R.W. Blamey[1] & R.I. Nicholson[2]

[1]*City Hospital, Nottingham;* [2]*Tenovus Institute, Cardiff, UK.*

**Summary** We have examined two new oestrogen receptor (ER) assays – an enzyme immunoassay (EIA) and an immunocytochemical assay (ICA) in a large series of primary breast tumours to compare their potential as predictors of (1) response to endocrine therapy and (2) survival in patients developing advanced breast cancer.

Response to endocrine therapy was categorised at 6 months (UICC criteria). ERICA appears the better predictor of response to endocrine therapy than ER-EIA. Combining ICA and EIA results did not improve the prediction of response.

With both assays patients with ER positive tumours survived longer from the time of diagnosis of advanced disease than those with ER negative tumours. The predictive power of these assay for progression of disease appears slightly better for the ER-ICA.

One measure of the clinical value of a hormone receptor assay in breast cancer is the accuracy with which it predicts response to systemic endocrine therapy. Response to therapy has been reported to correlate significantly with both oestrogen receptor (ER) status (Blamey *et al.*, 1980; Jensen, 1981; Stewart *et al.*, 1982; Williams *et al.*, 1986) and progesterone receptor (PgR) status (Stewart *et al.*, 1982; McGuire, 1978; Johnson *et al.*, 1983). Survival from commencing endocrine therapy has also been reported to correlate significantly with ER status (Hahnel *et al.*, 1979; Stewart *et al.*, 1981; Kinne *et al.*, 1981; Paterson *et al.*, 1982; Howat *et al.*, 1985; Williams *et al.*, 1987) and PgR status (Howat *et al.*, 1985; Howell *et al.*, 1984) of the primary tumour.

In recent years two new ER assays have become available from Abbott Laboratories an enzyme immunoassay (ER-EIA) and immunocytochemical assay (ER-ICA): both based on a monoclonal antibody (H222) that was raised to human ER. Although a previous study from our group has reported a statistically significant correlation between the ER-EIA and the ER-ICA in primary tumours (Walker *et al.*, 1988), to the authors knowledge no direct evaluation of the relative ability of these new assays to predict response to systemic endocrine therapy and survival from commencing treatment in patients with advanced breast cancer has been undertaken. This has been addressed in the current paper.

### Patients and methods

This study reports 192 patients with advanced breast cancer treated by primary endocrine therapy all of whom were assessable for response by International Union Against Cancer criteria (UICC) (Hayward *et al.*, 1977). Eighty-one patients received hormone therapy either for local disease (i.e. a locally advanced primary cancer or for recurrent local disease) while the remaining 111 patients had metastatic disease. The major sites of metastatic disease were bone only ($n = 41$), lung/soft tissue only ($n = 30$), bone and lung/soft tissue ($n = 12$) and visceral ($n = 28$).

All patients at initial presentation had primary tumour tissue available for oestrogen receptor measurement. Specimens consisted either of tumour biopsies removed in the out-patient clinic or surgical excision specimens. All specimens were immediately frozen in liquid nitrogens and maintained at a minimum of $-70°C$ until their assay in the Breast Cancer Unit of the Tenovus Institute for Cancer Research, Cardiff. Two types of oestrogen receptor assay were carried out using ER-EIA and ER-ICA kits obtained from Abbott Laboratories. Each assay has been described in detail (Nicholson *et al.*, 1986; Walker *et al.*, 1988) and will only be outlined here. The ER-EIA involved incubating cytosol fractions of the breast tumours with antibody (rat anti-human ER, D547) coated polystyrene beads. This immobilises the receptor prior to its incubation with a second antibody (rat anti-human ER H222) that has been conjugated with horseradish peroxidase. Following incubation and subsequent washing steps the concentration of horseradish peroxidase was determined using diaminobenzidine and $H_2O_2$ and is directly proportional to the concentration of ER. This provides a quantitative assessment of the receptor protein.

The ER-ICA procedure also utilises H222, but this time in a peroxidase – antiperoxidase (PAP) assay system. Briefly, H222 is incubated with a 5 $\mu m$ frozen section of the breast tumours' and after washing, this was followed by a bridging antibody (goat anti-rat IgG) and finally a rat PAP complex. Peroxidase activity is detected by the incubation of the antibody complex with diaminobenzidine and $H_2O_2$, with the insoluble reaction product marking the presence of the ER in the section.

In 98 patients oestrogen receptor status was measured by both the enzyme immunoassay (EIA) and immunocytochemical assay (ICA). In 80 patients oestrogen receptor status was measured using EIA alone and in 14 patients by ER-ICA alone. Therefore out of a total of 192 patients 178 had results for ER-EIA status and 112 patients had results for ER-ICA status.

In this study we have used a cut-off level of $<15$ fmol $mg^{-1}$ cytosol protein for the ER-EIA assay. Tumours were regarded as ER negative by the ER-ICA assay if $<5\%$ of tumour cells stained positive.

### Assessment

Patients have been assessed for complete or partial response (CR or PR), static disease (SD) and progression (PD) according to UICC criteria (Hayward *et al.*, 1977). As recommended by the British Breast Group (British Breast Group, 1974) it is our policy to assess patients for response and static disease 6 months after commencing hormone therapy.

All patients have been followed up for time to disease progression and for survival from commencement of primary hormone therapy.

Correspondence: J.F.R. Robertson, Professorial Unit of Surgery, City Hospital, Hucknall Road, Nottingham NG5 1PB, UK.
Received 24 May 1991; and in revised form 20 December 1991.

### Statistical analyses

Data were analysed using the statistical package SPSSX-21 (SPSS, 1986). Chi squared analysis with Yates correction where appropriate were used to compare frequencies of integers between two variables. Comparison of ER concentrations between groups was made using the Kruskal-Wallis test for non-parametric data. Survival between different groups was analysed using a modification of Gehan's generalised Wilcoxon test (Lee & Desu, 1972). In accordance with convention in all analysis $P<0.05$ was taken as significant.

### Results

Twenty-one patients showed a complete response and 21 a partial response at 6 months and 50 patients had static disease at 6 months. One hundred patients showed progression of disease within 6 months of commencing endocrine therapy.

ER expression was analysed to assess whether premenopausal and postmenopausal patients could be combined into a single group. Postmenopausal patients had significantly more ER positive tumours by ER-ICA (i.e. >5% tumour cells positive) than premenopausal patients ($P<0.03$; $\chi^2$ test). The concentration of ER in the tumour as determined by ER-EIA assay (fmoles $mg^{-1}$ cytosol protein) was also significantly higher in postmenopausal patients ($P<0.005$; Kruskal-Wallis test).

ER concentrations of the primary tumour was also correlated with the age of patients, based around the age limits of the United Kingdom Breast Cancer Screening Programme (i.e. 50–65 years). Patients were grouped as <50 years (i.e. premenopausal) or into one of two postmenopausal groups, 50–65 years or >65 years. Tumours were divided on the basis of the ER concentration by ER-EIA assay into <10, 11–100, 101–300 or >300 fmoles $mg^{-1}$ cytosol protein. ER expression correlated significantly with age (Table I) ($P=0.008$; $\chi^2$ test). Even between the two postmenopausal groups there were more patients with high ER concentration in the group of older patients (i.e. >65 years) (Table I).

We further examined the relationship between ER concentration and menopausal status to assess whether the same cut-off level was appropriate in premenopausal and postmenopausal patients in assessing response. A cut-off of 15 fmoles $mg^{-1}$ cytosol protein was selected and analysed to assess whether premenopausal and postmenopausal patients below or above this level had significantly different response rates. There was no difference using 15 fmoles $mg^{-1}$ cytosol protein as the cut-off. We repeated the analysis using 50 and 100 fmoles $mg^{-1}$ cytosol protein as cut-off levels and again found no difference in response rates between premenopausal and postmenopausal patients.

While the results above indicate that as a group postmenopausal patients have significantly higher levels of ER expression in their tumours these results also show that tumours with equal concentrations of ER show similar response rates irrespective of menopausal status of patients. Since as a group postmenopausal patients have higher concentrations of ER the response rate in a group of postmenopausal patients would be expected to exceed that found in a group of premenopausal patients. However this difference in response rate by menopausal status failed to reach statistical significance ($P=0.09$; $\chi^2$ test).

In view of the above results showing that the same cut-off levels for ER expression can be used in all patients, we combinated premenopausal and postmenopausal patients into a single group. ER concentration by ER-EIA assay was correlated with the site of initial disease (Figure 1). There was a significant difference in ER concentration between patients with local disease and patients with metastatic disease ($P<0.02$; Kruskal Wallis test). Further analyses showed that the only significant difference was between patients with local disease and patients with lung metastases ($P<0.002$; Kruskal Wallis test). This difference in ER concentration would explain the significant difference in response rates between different sites of initial disease: patients with local disease had a significantly higher response rate than patients with metastatic disease ($\chi^2=53.9$; 1 d.f.: $P<0.0001$).

**Table I** Patient age *vs* ER expression (ER-EIA assay)

| *ER concentration (fmoles $mg^{-1}$ protein)* | *Age (years)* <50 | 50–65 | >65 |
|---|---|---|---|
| <10 | 19 | 21 | 17 |
| 11–100 | 26 | 21 | 16 |
| 101–300 | 7 | 19 | 9 |
| >300 | 1 | 7 | 12 |

$\chi^2=17.3$, 6 d.f.; $P=0.008$.

**Figure 1** ER by site of initial disease.

Response to treatment and survival was compared separately with hormone receptor status measured by ER-EIA and ER-ICA.

### *ER-EIA status*

One hundred and seventy-eight patients had ER-EIA receptor status measured. At presentation 49 were premenopausal and 129 postmenopausal. The main site of disease on commencing hormone therapy was local disease ($n=72$), bone metastasis only ($n=38$), lung/soft tissue metastasis ($n=29$), bone and lung/soft tissue metastasis ($n=12$) and visceral metastasis ($n=27$). One hundred and thirty-two patients were treated with tamoxifen, 20 mg b.d., 39 patients with goserelin (Zoladex, ICI Pharmaceuticals, UK), 3.6 mg by monthly subcutaneous injection alone or in combination with tamoxifen and seven patients with megestrol acetate (Megace, Bristol Myers, UK), 160 mg b.d. ER-EIA receptor status correlated significantly with UICC assessed response at 6 months as shown in Table II. ER-EIA status also correlated significantly with time to progression of disease (Wilcoxon statistic = 27.7, 1 d.f.; $P<0.0001$), (Figure 2) and with survival (Wilcoxon statistic = 26.5, 1 d.f. $P<0.0001$), (Figure 3): patients with ER-EIA positive tumours have a much more favourable outlook.

### *ER-ICA status*

One hundred and twelve patients had ER-ICA receptor status measured, 27 premenopausal and 85 postmenopausal. The main site of disease on commencing hormone treatment was local disease ($n=60$), bone metastasis only ($n=18$), soft

**Table II** UICC assessed response to primary hormone therapy by ER status of the primary tumour

| | *CR* | *PR* | *Static* | *Prog.* | |
|---|---|---|---|---|---|
| *ER-EIA* | | | | | |
| − | 1 | 4 | 10 | 50 | $\chi^2 = 23.6$, 3 d.f.; |
| + | 17 | 11 | 39 | 46 | $P<0.0001$ |
| *ER-ICA* | | | | | |
| − | 0 | 0 | 6 | 35 | $\chi^2 = 38.4$, 3 d.f.; |
| + | 14 | 15 | 23 | 19 | $P<0.0001$ |

CR = complete response; PR + partial response; Static = static disease; Prog. = progressive disease.

**Figure 2** Probability of progression by ER status. —□—, ER-EIA positive; —■—, ERICA positive; —◆—, ER-EIA negative; —◇—, ERICA negative.

**Figure 3** Probability of survival by ER status. —□—, ER-EIA positive; —■—, ERICA positive; —◆—, ER-EIA negative; —◇—, ERICA negative.

tissue/lung metastasis ($n = 14$), bone and soft tissue/lung metastasis ($n = 5$) and visceral metastasis ($n = 15$). Eight-seven patients were treated with tamoxifen, 22 patients with goserelin alone or in combination with tamoxifen and three patient with megestrol acetate. ER-ICA receptor status correlated significantly with UICC assessed response at 6 months, as shown in Table II. ER-ICA status also correlated significantly with time to progression of disease (Wilcoxon statistic = 33.3, 1 d.f.; $P<0.0001$), (Figure 2) and with survival (Wilcoxon statistic = 31.0, 1 d.f.; $P<0.0001$), (Figure 3).

### *ER-EIA and ER-ICA status combined*

Ninety-eight patients had both ER-EIA and ER-ICA status measured. Comparison of combined ER-EIA/ER-ICA status *vs* UICC response at 6 months is shown in Table III. Combining ER-ICA and EIA results did not appear to improve the prediction of response to endocrine therapy *vs* either ER-EIA or ER-ICA status alone (Table III).

**Table III** UICC assessed response to primary hormone therapy by ER-EIA/ER-ICA of the primary tumour combined

| | *CR* | *PR* | *Static* | *Prog.* |
|---|---|---|---|---|
| *ER-ICA/ER-EIA* | | | | |
| + + | 10 | 9 | 22 | 17 |
| + − | 1 | 0 | 0 | 1 |
| − + | 0 | 0 | 3 | 9 |
| − − | 0 | 0 | 3 | 23 |

$\chi^2 = 33.9$, 9 d.f.; $P<0.0001$.

Both assays were assessed for sensitivity in predicting response (CR + PR) and specificitiy in predicting non-response (SD + PD): the results were 28/33 (85%) and 60/145 (41%) respectively for ER-EIA and 29/29 (100%) and 41/83 (49%) for ERICA. The assays were further evaluated for sensitivity in predicting non-progression (CR + PR + SD) and specificity for progression (PD): the results for ER-EIA were 67/82 (82%) and 50/96 (52%) respectively and for ERICA 52/58 (90%) and 35/54 (65%). In both analyses, sensitivity and specificity appeared better by ERICA than ER-EIA.

### Discussion

Results obtained from hormone receptor assays must be both specific (able to predict failure) and sensitive (able to predict response) in order to provide useful prognostic information. It has previously been reported that using the ligand binding assay 32% of patients with ER positive primary tumours responded (CR + PR/total) to systemic endocrine therapy for recurrent carcinoma compared to 10% of patients with ER negative tumours (Williams *et al.*, 1987). Similar response rates of 25% and 8% in patients with ER positive and negative tumours respectively, as measured by the ER-EIA assay, are reported in this paper. Patients with ER-ICA positive or negative tumours showed 41% or 0% response rates respectively. Similar response rates for ER-ICA positive and negative tumours (39% and 0% respectively) have been reported (McClelland *et al.*, 1986). This present study supports these results and suggests that the sensitivity and specificity of the ER-ICA assay for predicting response/non-respnse or progression/non-progression is better than the ER-EIA assay.

This study found that ER expression in primary tumours was significantly higher in the postmenopausal group of patients. The expression of ER correlated with the age of patients at initial presentation with breast cancer. The higher concentrations of ER with increasing age was also seen between the two postmenopausal age groups (i.e. 50–65 and >65 years). These latter results suggest that the correlation of menopausal status with ER expression may simply be a reflection of the relationship between ER expression and patient age. High levels of ER expression in the primary tumours of elderly patients has previously been reported (Allan *et al.*, 1985; Legha *et al.*, 1978).

Survival from commencing endocrine therapy has previously been reported to correlate significantly with ER status by the ligand binding assay (Williams *et al.*, 1986). Survival from commencing endocrine therapy by ER status of the primary tumour was significantly longer from patients with ER positive tumours measured both by ER-EIA and ER-ICA (Figure 3). The difference in survival between ER positive and ER negative patients was equally well shown by ER-ICA and ER-EIA.

Walker and colleagues have previously reported a good correlation between ER status of the primary tumour as measured by ER-EIA and ER-ICA (Walker *et al.*, 1988). This correlation between these two ER assays was confirmed in this study in that there was agreement on the ER status in 84/98 (85%) of patients (Table III). Coombes and colleagues have reported that ER status measured by ER-ICA is a good predictor of response to therapy (Coombes *et al.*, 1987). This paper comparing the value of ER-ICA and ER-EIA assays in patients with advanced breast cancer extends the observations regarding ER-ICA by suggesting it is as good a predictor of survival as ER-EIA which requires solubilisation of a larger portion of tumour tissue. In addition ER-ICA status has higher sensitivity and specificity than ER-EIA status in predicting both therapeutic response and progression of disease on primary endocrine therapy.

The ER-ICA assay has several other distinct advantages. Firstly ER-ICA can be performed on a small tumour biopsy sample or even an aspirate (Coombes *et al.*, 1987). Secondly it provides information on the number of tumour cells expressing ER and between tumour tissue and its benign components. Since a previous study from our group reported that response rates improved with increasing concentration of ER estimated by the ligand binding method (Campbell *et al.*, 1981) the heterogeneity of ER expression by tumour cells noted on ER-ICA examination is currently the subject of further research to try and establish the predictive value, if any, of the % of tumour cells which stain positive both in primary operable breast cancer and in advanced breast cancer.

### References

ALLAN, S.G., RODGER, A., SMYTH, J.F., LEONARD, R.C.F., CHETTY, U. & FORREST, A.P. (1985). Tamoxifen as primary treatment of breast cancer in elderly or frail patients: a practical management. *Br. J. Med.*, **290**, 358.

BLAMEY, R.W., BISHOP, H.M., BLAKE, J.R.S. & 5 others (1980). Relationship between primary breast tumour receptor status and patient survival. *Cancer*, **46**, 2765–2769.

BRITISH BREAST GROUP (1974). Assessment of response to treatment in advanced breast cancer. *Lancet*, **ii**, 38–39.

CAMPBELL, F.C., BLAMEY, R.W., ELSTON, C.W. & 4 others (1981). Quantitative oestradiol receptor values in primary breast cancer and response in metastases to endocrine therapy. *Lancet*, **ii**, 1317.

COOMBES, R.C., POWLES, T.J., BERGER, U. & 5 others (1987). Prediction of endocrine response in breast cancer by immunocytochemical detection of oestrogen receptor in fine-needle aspirates. *Lancet*, **ii**, 701–703.

HAHNEL, R., WOODINGS, T. & VIVIAN, A.B. (1979). Prognostic value of oestrogen receptors in primary breast cancer. *Cancer*, **44**, 671–675.

HAYWARD, J.L., CARBONE, P.P., HEWSON, J.C., KUMAOKA, S., SAGALOF, A. & RUBENS, R.V. (1977). Assessment of response to therapy in advanced breast cancer. *Cancer*, **39**, 1289–1294.

HOWAT, J.M.T., HARRIS, M., SWINDELL, R. & BARNES, D.M. (1985). The effect of oestrogen and progesterone receptors on recurrence and survival in patients with carcinoma of the breast. *Br. J. Cancer*, **51**, 236–270.

HOWELL, A., HARLAND, R.N.L., BRAMWELL, V.H.C. & 6 others (1984). Steroid hormone receptors and survival after first relapse in breast cancer. *Lancet*, **i**, 588–591.

JENSEN, E.V. (1981). Hormone dependency of breast cancer. *Cancer*, **47**, 2319–2326.

JOHNSON, P.A., BONOMI, P.D., ANDERSON, K.M. & 4 others (1983). Progesterone receptor as a predictor of response to megestrol acetate in advanced breast cancer: a retrospective study. *Cancer Treat. Rep.*, **67**, 717–720.

KINNE, D.W., ASHIKARI, R., BUTLER, A., MENENDEZ-BOTET, C., ROSEN, P.P. & SCHWARTZ, M. (1981). Estrogen receptor protein in breast cancer as a predictor of recurrence. *Cancer*, **47**, 2364–2367.

LEE, E.T. & DESU, M.M. (1972). A computer program for comparing κ samples with right censored data. *Computer Programmes Biomed.*, **2**, 315–321.

LEGHA, S.S., DAVIS, H.L. & MUGGIA, F.M. (1978). Hormonal therapy of breast cancer: new approaches and concepts. *Ann. Intern. Med.*, **88**, 69–77.

MCCLELLAND, R.A., BERGER, U., MILLER, L.S., POWLES, T.J. & COOMBES, R.C. (1986). Immunocytochemical assay for estrogen receptor in patients with breast cancer: relationship to a biochemical assay and to outcome of therapy. *J. Clin. Oncol.*, **4**, 1171–1176.

MCGUIRE, W.L. (1978). Hormone receptors: their role in predicting prognosis and response to endocrine therapy. *Seminars in Oncol.*, **5**, 428–433.

NICHOLSON, R.I., COLIN, P., FRANCIS, A.B. & 6 others (1986). Evaluation of an enzyme immunoassay for oestrogen receptors in human breast cancers. *Cancer Res. (Suppl)*, **46**, 4299s–4230s.

PATERSON, A.H.G., ZUCK, V.P., SZAFRAN, O., LEES, A.W. & HANSON, J. (1982). Influence and significance of certain prognostic factors on survival in breast cancer. *European J. Cancer Clin. Oncol.*, **18**, 937–943.

SPSS INC. (1986). *SPSS$^x$ User's Guide*, McGraw-Hill: New York.

STEWART, J.F., KING, R.J.B., SEXTON, S.A., MILLIS, R.R., RUBENS, R.D. & HAYWARD, J.L. (1981). Oestrogen receptors, site of metastatic disease and survival in recurrent breast cancer. *Europ. J. Cancer*, **17**, 449–453.

STEWART, J.F., KING, R., HAYWARD, J.L. & RUBENS, R.D. (1982). Estrogen and progesterone receptors. Correlation of response rates, site and timing of receptor analysis. *Breast Cancer Res. & Treat.*, **2**, 243–250.

WALKER, K.J., BOUZABAR, N., ROBERTSON, J. & 6 others (1988). Immunocytochemical localisation of estrogen receptor in human breast tissue. *Cancer Res.*, **48**, 6517–6522.

WILLIAMS, M.R., TODD, J.H., NICHOLSON, R.I., ELSTON, C.W., BLAMEY, R.W. & GRIFFITHS, K. (1986). Survival patterns in hormone treated advanced breast cancer. *Br. J. Surg.*, **73**, 752–755.

WILLIAMS, M.R., TODD, J.H., ELLIS, I.O. & 6 others (1987). Oestrogen receptors in primary and advanced breast cancer: an eight year review of 704 cases. *Br. J. Cancer*, **55**, 67–73.